# Synergistic effects of the Aβ/fibrinogen complex on synaptotoxicity, neuroinflammation, and blood–brain barrier damage in Alzheimer's disease models

**DOI:** 10.1002/alz.70119

**Published:** 2025-05-08

**Authors:** Elisa Nicoloso Simões‐Pires, Daniel Torrente, Pradeep Singh, Sidney Strickland, Erin H. Norris

**Affiliations:** ^1^ Patricia and John Rosenwald Laboratory of Neurobiology and Genetics The Rockefeller University New York New York USA

**Keywords:** Alzheimer's disease, amyloid‐beta, blood‐brain barrier, fibrinogen, neuroinflammation, synaptotoxicity

## Abstract

**INTRODUCTION:**

Alzheimer's disease (AD) is characterized by amyloid‐beta (Aβ), hyperphosphorylated tau, chronic neuroinflammation, blood–brain barrier (BBB) damage, and synaptic dysfunction, leading to neuronal loss and cognitive deficits. Vascular proteins, including fibrinogen, extravasate into the brain, further contributing to damage and inflammation. Fibrinogen's interaction with Aβ is well‐established, but how this interaction contributes to synaptic dysfunction in AD is unknown.

**METHODS:**

Organotypic hippocampal cultures (OHC) were exposed to Aβ42 oligomers, fibrinogen, or Aβ42/fibrinogen complexes. Synaptotoxicity was analyzed by Western blot. Aβ42 oligomers, fibrinogen, or their complexes were intracerebroventricularly injected into mice. Histopathological AD markers, synaptotoxicity, neuroinflammation, and vascular markers were observed by Western blot and immunofluorescence.

**RESULTS:**

Aβ42/fibrinogen complexes led to synaptic loss, tau181 phosphorylation, neuroinflammation, and BBB disruption, independent of Mac1/CD11b receptor signaling. Blocking Aβ42/fibrinogen complex formation prevented synaptotoxicity.

**DISCUSSION:**

These findings indicate that the Aβ42/fibrinogen complex has a synergistic impact on hippocampal synaptotoxicity and neuroinflammation.

**Highlights:**

- Fibrinogen binds to the central region of Aβ, forming a plasmin‐resistant complex.
- The Aβ/fibrinogen complex induces synaptotoxicity, inflammation, and BBB disruption.
- Synaptotoxicity induced by the complex is independent of Mac1 receptor signaling.

## 1 BACKGROUND

Alzheimer's disease (AD) is a neurodegenerative disorder that leads to significant memory loss. The major histopathological hallmarks of AD are the abundant inclusions of hyperphosphorylated tau and amyloid‐beta (Aβ) in the brain. The importance of Aβ in AD pathogenesis is strongly supported by evidence that mutations in amyloid precursor protein (APP) or presenilins (PSENs), proteins that are responsible for the generation of Aβ peptides, lead to familial AD with abundant Aβ deposits and tau inclusions.^1^, ^2^, ^3^ The fact that tau inclusions arise with APP or PSEN mutations suggests that these proteinopathies are linked. Various other neuropathological deficits include neurodegeneration, neuroinflammation, and blood–brain barrier (BBB) damage.^4^, ^5^, ^6^

Late‐onset AD accounts for 95% of AD cases.^7^, ^8^ Soluble oligomeric/protofibrillar forms of Aβ42 are thought to be the most toxic species of this pathogenic peptide.^9^

The contribution of inflammation to the pathophysiology of AD has been extensively explored. Notably, neuroinflammation is characterized by reactive gliosis surrounding Aβ plaques and neurofibrillary tangles (NFTs). Persistent glial activation and the generation of pro‐inflammatory cytokines can trigger neurodegenerative mechanisms due to the subsequent loss of synapses and neurons. Activated microglia may act as a starting point for the observed neurodegeneration in AD pathology.^5^, ^10^, ^11^ Moreover, the BBB is an essential physical barrier that protects the brain from external material interference, and its integrity affects the process of AD. It relies on astrocytic processes, neurons, and microglia, which collectively form the neurovascular unit.^12^, ^13^ Human studies have demonstrated increased BBB permeability in individuals with dementia as compared to those without.^14^

Fibrinogen, a vital blood coagulation protein, is normally excluded from the brain parenchyma by the BBB.^15^ Recent findings suggest that fibrinogen could serve as a molecular link between neurovascular disruption, neuroinflammation, and neurological impairment in AD patients. The fibrinogen *γ*‐chain was found in the cerebrospinal fluid (CSF) of AD patients but not in other neurodegenerative conditions, identifying fibrinogen as a distinct biomarker for AD.^16^, ^17^, ^18^ Consistent with these human data, we and others have shown that fibrinogen extravasates from the blood vessels into the brain parenchyma and co‐localizes with Aβ plaques, dystrophic neurites, and activated microglia in multiple animal models of AD.^19^, ^20^, ^21^, ^22^, ^23^ We have previously shown that fibrinogen interacts with Aβ, leading to fibrinolysis‐resistant clots in the brain and around blood vessels.^24^, ^25^, ^26^, ^27^ More recently, we showed that the United States Food and Drug Administration (FDA) ‐approved AD immunotherapy, lecanemab, can block and dissociate Aβ42/fibrinogen complexes, therefore improving fibrinolysis and clot abnormalities in human plasma ex vivo and preventing synaptotoxicity in vitro.^28^ While the interaction between Aβ42 and fibrinogen is well recognized, the extent by which vascular damage contributes to synaptic dysfunction and how it collaborates with amyloid pathology to induce neuroinflammation and cognitive decline remains poorly understood.

In this study, we investigated the potential of the Aβ42/fibrinogen complex to induce synaptotoxicity and activate inflammatory cells. We also examined the subsequent effects of Aβ42/fibrinogen complex formation on early‐stage AD‐like progression in mice. We found that low concentrations of these proteins alone do not induce neurotoxic deficits, but low doses of Aβ42/fibrinogen complexes have a synergistic negative effect on synapses in vitro. This effect was blocked when blocking the Aβ42/fibrinogen interaction. Moreover, we show that the Aβ42/fibrinogen complex induced synapse protein loss, BBB damage, and blood protein extravasation into the hippocampus of wild‐type (WT) mice.

**RESEARCH IN CONTEXT**

1. Systematic review: Literature was reviewed by means of traditional sources (e.g., PubMed). Increasing evidence points to a role for vascular dysfunction in Alzheimer's disease (AD) pathophysiology. AD patient brains show fibrin deposition in areas associated with cell death, and amyloid‐beta (Aβ) binds to fibrin(ogen), leading to clots that are structurally abnormal and degradation‐resistant. The mechanisms by which fibrinogen contributes to AD pathology are still limited. Here we used mouse systems to determine the effect the Aβ/fibrinogen complex on AD pathology.
2. Interpretation: We show that the Aβ/fibrinogen interaction has a synergistic effect on hippocampal synaptotoxicity and neuroinflammation, independent of Mac1/CD11b receptor‐mediated signaling and blood‐brain barrier disruption. These results enhance our understanding of how vascular dysfunction can impact AD pathophysiology.
3. Future directions: Further research is needed to determine the precise cellular and molecular mechanisms by which the Aβ/fibrinogen complex negatively impacts AD pathogenicity. A better understanding of these mechanisms could lead to new diagnostic strategies and therapies.

The Aβ42/fibrinogen complex also induced astrogliosis and activation of microglia/infiltrating monocytes and macrophages in this in vivo model, and these neuroinflammatory responses were independent of microglial Mac‐1/CD11b receptor signaling.

## 2 METHODS

### 2.1 Reagents

Plasma‐purified human fibrinogen was obtained from EMD Millipore. Aβ42 peptide was from Bachem. Purified anti‐Aβ 17‐24 antibody (4G8) was from BioLegend, and anti‐Aβ 1‐5 antibody (mOC64) was from Abcam. 1,1,1,3,3,3‐Hexafluoro‐2‐propanol (HFIP), purchased from Sigma, was used to prepare Aβ42 oligomers (see below).

### 2.2 Animals

C57BL/6 and B6.129S4‐*Itgamtm1Myd*/J (Mac‐1 knockout [KO]) mice were purchased from Jackson Laboratory and bred and maintained in our lab. Mouse strains were housed with food and water ad libitum, under controlled temperature (20°C–22°C), humidity (40%–60%), and illumination (12/12‐h dark cycle). All animal experiments were conducted in accordance with the ARRIVE guidelines, the US National Institutes of Health (NIH) Guide for the Care and Use of Laboratory Animals, and with approval from the Animal Care and Use Committee of The Rockefeller University.

### 2.3 Organotypic hippocampal slice cultures

Organotypic hippocampal slice cultures (OHCs) were prepared according to Stoppini and coworkers^29^ with slight modifications.^30^ Briefly, post‐natal day 8‐10 C57BL/6 or Mac‐1 KO mice, when indicated, were decapitated instantaneously, and their brains were quickly removed from the skull and washed with ice‐cold Hanks’ balance salt solution (HBSS, Gibco). Both hippocampi were dissected and cut into 400 µm‐thick slices using a McIlwain tissue chopper (Mickle Laboratories). Slices were separately immersed into ice‐cold HBSS and arranged onto organotypic inserts of standard six‐well cell culture plates (Millicell, Millipore). Each organotypic insert contained six hippocampal slices total from three distinct animals (two slices per animal), and the result from each insert generated a single data point within a single experiment. Three to five separate inserts were used for each treatment group. Each independent experiment consisted of three animals, and each data point represents the average/combined result of three mice. The numbers of independent experiments (*n*) are indicated in the legend of each figure. The slices were maintained in culture in an incubator at 37°C and 5% CO2 in air using an interface method with 1 mL medium supplying the undersurface of the insert. The culture medium consisted of 50% minimum essential medium (Gibco), 25% HBSS (Gibco), 25% heat‐inactivated horse serum (Gibco), supplemented with 36 mM D‐glucose (Sigma), 25 mM HEPES (Sigma), 4 mM NaHCO3 (Fisher Scientific), and 1% penicillin/ streptomycin/ amphotericin (Gibco), pH 7.3. The first medium change was made 30 min following preparation and repeated every 3–4 days. Slices were maintained for 14 days in vitro (DIV) prior to experiments.

### 2.4 Preparation of and treatment with Aβ42 oligomers and their complex with fibrinogen

Aβ42 oligomers were prepared as described^9^ with slight modifications.^26^, ^30^ Briefly, Aβ42 was resuspended in ice‐cold HFIP and dried to form peptide films. For Aβ42 aggregate preparation, Aβ42 films were dissolved in dimethyl sulfoxide (DMSO), sonicated, and diluted in ice‐cold phosphate buffered saline (PBS), pH 7.4. The final concentration of DMSO was 5%. The samples were incubated for 24 h at 4°C in Protein LoBind tubes (Eppendorf). After incubation, the samples were centrifuged at 20,000 x *g* for 10 min at 4°C, and the supernatant, which contained soluble Aβ42 aggregates, was collected. The peptide concentration was determined using Pierce bicinchoninic acid (BCA) protein assay kit (Thermo Scientific). Purified human fibrinogen was prepared in PBS (pH 7.4) as described.^26^

At 14 DIV, Aβ42 and fibrinogen solutions were prepared. OHCs as described above were exposed for 24 h at 37°C and 5% CO2 to Aβ42 oligomers (150 or 500 nM), fibrinogen (50 nM), Aβ42 and fibrinogen (150 nM Aβ + 50 nM fibrinogen), or an equivalent volume of vehicle containing 5% DMSO in PBS. The Aβ42 and/or fibrinogen solutions were added to 1 mL culture medium to the final concentration to each separate Eppendorf tube for 1 h with gentle agitation at 37°C prior to exposure. Aβ42/fibrinogen complex formation occurs during this 1 h incubation time. To block the Aβ42/fibrinogen interaction, Aβ42 (150 nM) and the 4G8 or mOC64 anti‐Aβ antibody (150 nM; 1:1 ratio) were incubated in 1 mL culture media for 30 min with gentle agitation at 37°C. Following Aβ42's incubation with 4G8 or mOC64, fibrinogen (50 nM) was added and the mixture was further incubated for 1 h with gentle agitation at 37°C. After both incubation periods, the mixture was added to the culture and incubated as described above.

For the in vivo studies, Aβ42 oligomers, the Aβ42/fibrinogen pre‐formed complex, and [(Aβ42/4G8) + fibrinogen] solutions were prepared as described above. The final concentration of these solutions was diluted in artificial cerebrospinal fluid (aCSF) followed by 1 h with gentle agitation at 37°C prior to surgeries.

### 2.5 Intracerebroventricular injections

Male C57BL/6 or Mac‐1 KO mice at postnatal day 90 were deeply anesthetized using a mixture of oxygen and isoflurane and had anesthesia sustained by a mask attached to the system. Following shaving of the head, disinfection of the skin, and local subcutaneous (s.c.) injection of meloxicam SR (6 mg/kg), an incision was made to expose the skull. The skull was thoroughly dried by removing all bone‐attached membrane and fat tissue, and a hole on the right side was opened using a dental drill. We stereotactically injected Aβ42 oligomers (1.5 or 3.0 µM), the pre‐formed Aβ42/fibrinogen complex (1.5 µM 156 + 7.5 µM), fibrinogen (7.5 µM), or aCSF into the right lateral ventricle (anteroposterior [AP], −0.2 mm; mediolateral [ML], 1.0 mm; dorsoventral, −2.4 mm from bregma,^11^ according to Paxinos and Franklin). All solutions were slowly injected (500 nL/min) with a 2.5 µL Hamilton syringe attached to a 33‐gauge needle and left in place for 5 min. Nylon sutures were used to close the incision site, and s.c. meloxicam S.R. (6 mg/kg) was administered every 2 days until perfusion and tissue collection.

### 2.6 Western blot

To estimate protein content for the in vitro model, independent experiments were carried out with OHCs prepared as described above. At the end of each experiment, the slices were rinsed with PBS, lysed in ice‐cold RIPA buffer containing 25 mM Tris‐HCl (pH 7.6), 150 mM NaCl, 1% NP‐40, 1% sodium deoxycholate, 0.1% sodium dodecyl sulfate (SDS), and protease inhibitor and phosphatase inhibitor cocktails (Thermo Scientific), and homogenized by vigorous vortex shaking. For the in vivo experiments, samples of whole contra‐lateral hippocampal homogenates were taken from adult animals after perfusion and quick dissection of hippocampus on an ice‐cold surface. The same solution used for hippocampal cultures was used for the homogenization of the whole hippocampus. Samples were then centrifuged at 12,000 x *g* for 15 min at 4°C. Protein concentration was estimated by the BCA assay (Thermo Scientific). Protein (30 µg) was loaded into each lane of 4‐20% SDS‐polyacrylamide gel electrophoresis (SDS‐PAGE) gels (Bio‐Rad), transferred to polyvinylidene fluoride (PVDF) membranes (Bio‐Rad), and processed. The membranes were blocked with blocking buffer (Bio‐Rad) and incubated overnight at 4°C with primary antibodies: anti‐synaptophysin (SYP, 1:2000, Abcam #ab8049), anti‐PSD‐95 (1:2000, Abcam #ab18258), anti‐p‐tau181 (1:1000, Cell Signaling #12885S), anti‐tau (1:1000, Cell Signaling #46687S), anit‐CD31 (1:1000, Abcam #ab124432), occludin (1:1000, Thermo Scientific #71‐1500), ZO‐1 (1:1000, Proteintech #21773‐1‐AP), AQP‐4 (1:1000, Sigma #A5971), and anti‐*β‐*actin (1:20,000, Sigma #A5441). Each Western blot membrane was separately probed with several primary antibodies against different molecular weight proteins without needing to strip the membrane. Therefore, the same *β‐*actin bands are shown in multiple figures (Figure 2A and Figure 5F; Figure S4). Washed membranes were incubated with horseradish peroxidase (HRP)‐conjugated secondary antibodies for 2 h and visualized with ECL reagent (Bio‐Rad). The membranes were imaged on a ChemiDoc Imaging System (Bio‐Rad), and optical density was estimated with NIH ImageJ software. Protein immunoreactivities were normalized with respect to *β*‐actin loading control.

### 2.7 Immunofluorescence

Three days post‐surgery, mice were deeply anesthetized by intraperitoneal (IP) injection of 1.5% tribromoethanol and perfused with 0.9% ice‐cold saline containing 2.5% heparin. For fixed brain tissue, brains were removed, and the right hemisphere was fixed in 4% paraformaldehyde^31^ in 0.1 M phosphate buffer^32^ for 24 h at 4°C. Brains were then dehydrated in sucrose gradient (10%, 20%, 30%) in PBS at 4°C for 24 h before being processed for optimal cutting temperature^33^ embedding and stored at −80°C until used. Brains were sectioned at a thickness of 25 µm using a cryostat, and sections were collected into PBS, transferred into cryoprotectant (30% glycerol, 30% ethylene glycol, 40% 0.1 M PB), and stored at −20°C until later use. For analysis, sections were then placed in a 24‐well plate and washed three times with PBS, and immunolabeling was performed on free‐floating sections. All sections were blocked in 10% normal donkey serum with 0.5% Triton X‐100 for 2 h at room temperature (RT). Primary antibodies were diluted in 5% normal donkey serum with 0.3% Triton X‐100, and sections were incubated with primary antibody overnight at 4°C. The following antibodies and dilutions were used: anti‐glial fibrillary acidic protein (GFAP) (1:1000, Sigma #3670S) and anti‐CD68 (1:500, Bio‐Rad #MCA1957GA). The following day, sections were incubated with an appropriate secondary antibody (1:1000, Alexa Fluor, donkey anti‐host species, Life Technologies) for 2 h at RT. Coverslips were added using Vectashield Mounting Medium with 4′,6‐diamidino‐2‐phenylindole (DAPI; Vector). Images were obtained on a confocal microscope (LSM 780, Zeiss) with a 20×/1.3 NA objective under fixed imaging settings. Stacks of at least 10 equidistant (250 nm) planes were taken from each tissue section and analyzed for each animal. Maximum projections were obtained from those *z*‐stacks and corrected for photobleaching by normalizing the signal to the average signal of each slice. Images were thresholded based on control slices immunostained using secondary antibodies alone run in parallel with each experiment. Intensity of fluorescence was measured with NIH ImageJ software. Four sections per mouse were analyzed, focusing on the CA1 region of the hippocampus.

### 2.8 Statistical analyses

All statistical analyses were performed using GraphPad Prism 10.0 software. Comparisons among multiple groups were performed using one‐way analysis of variance (ANOVA) followed by Tukey's multiple comparison test. Power analyses were based on prior publications by the first author, E. Nicoloso Simões‐Pires.

## 3 RESULTS

### 3.1 The Aβ42/fibrinogen complex induces synergistic synaptotoxicity in organotypic mouse hippocampal culture

**FIGURE 1:**
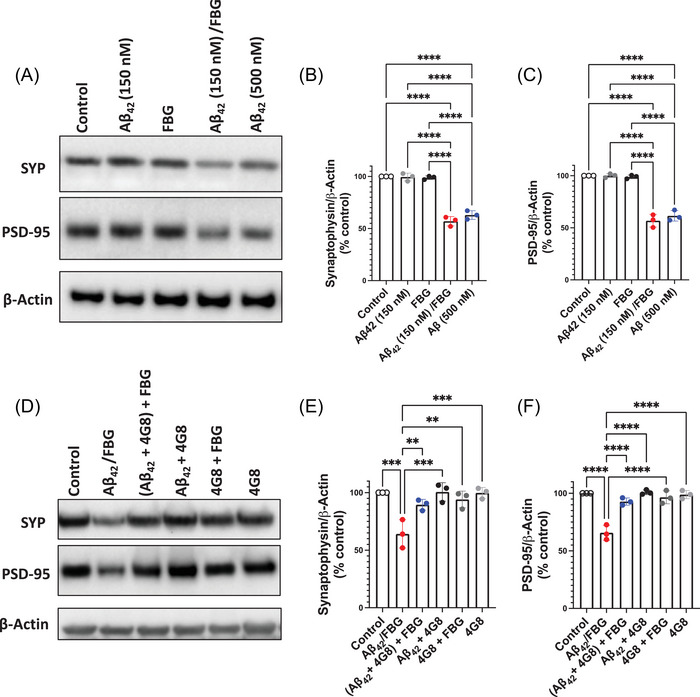
The Aβ_42_/fibrinogen complex induces synergistic synaptotoxicity in mouse organotypic hippocampal slice cultures (OHCs). (A)–(C) OHCs were incubated with vehicle (control; 0.005% DMSO in PBS), low‐dose Aβ42 oligomers (150 nM), high‐dose Aβ42 oligomers (500 nM), fibrinogen (FBG, 50 nM), or Aβ42/FBG complex (150 and 50 nM, respectively) for 24 h and then collected for biochemical analysis. (A) Representative Western blot of SYP and post‐synaptic density 95 (PSD‐95) levels in OHC lysates. Protein analyses were normalized to *β*‐actin. Quantification shows a reduction in (B) SYP and (C) PSD‐95 in OHCs treated with the Aβ42/FBG complex (red) or the high dose of Aβ alone (blue). (D)–(F) OHCs were treated with vehicle (control), low‐dose Aβ42 oligomers (150 nM), FBG (50 nM), or Aβ42/FBG complex with or without 4G8 anti‐Aβ antibody for 24 h and then collected and prepared for biochemical analysis. (D) Representative Western blot of SYP and PSD‐95 levels in OHC lysates. Quantification shows a reduction of (E) SYP and (F) PSD‐95 in OHCs treated with Aβ42/FBG complex only (red). Slices treated with Aβ42/fibrinogen and 4G8 (blue) showed no changes in synapse protein levels. *n* = 3 independent experiments. More precisely, each well/insert contained six hippocampal slices total from three different animals (two slices per animal), and there were three wells per treatment group. The result from each well generated a single data point, although it represented three animals. Each experiment encompassed nine mice but only three data points. Data were analyzed by one‐way ANOVA with Tukey's post hoc analysis. Aβ, amyloid‐beta; ANOVA, analysis of variance; DMSO, dimethyl sulfoxide; PBS, phosphate buffered saline; SYP, synaptophysin. ^**^ *p* < 0.01, ^***^ *p* < 0.001, ^****^ *p* < 0.0001

The hippocampus plays a crucial role in learning and memory. Disruptions in hippocampal synaptic function correspond with neuropathological alterations and the progression of dementia in AD.^34^, ^35^, ^36^ To investigate the effects of Aβ42 oligomers with and without fibrinogen on hippocampal synaptotoxicity, we used an organotypic hippocampal culture (OHC) system, which fully preserves tissue integrity and its cellular components, prepared from WT mice.^30^ AD synaptotoxicity was mimicked in vitro by exposing OHCs to varying doses of Aβ42 oligomers in the presence or absence of fibrinogen, and the levels of synaptic proteins were assessed by Western blot. Synaptophysin (SYP) and postsynaptic density protein 95 (PSD‐95) are critical elements of synapses, and their alterations are associated with synaptic dysregulation, which correlates with cognitive decline in AD.^37^, ^38^ OHCs treated with a low dose of Aβ42 oligomers (150 nM) or fibrinogen (50 nM) showed no changes in SYP and PSD‐95 protein levels, while a high dose of Aβ42 oligomers (500 nM) led to decreased levels of both synaptic proteins as previously shown.^26^, ^30^ Notably, OHC treatment with the preformed Aβ42/fibrinogen complex, comprised of low‐dose Aβ42 oligomers (150 nM) and fibrinogen (50 nM), exhibited a significant decrease in SYP and PSD‐95 (Figure 1A–C; red vs. open circles).

Synaptotoxicity induced by the Aβ42/fibrinogen complex was similar to that from high‐dose Aβ oligomers (500 nM; Figure 1AC). These data suggest that the Aβ42/fibrinogen complex induces a synergistic synaptotoxic effect on WT mouse OHCs.

To confirm that the observed synaptotoxicity was indeed driven by the Aβ42/fibrinogen complex, we pre‐incubated Aβ42 oligomers with anti‐Aβ monoclonal antibodies targeting different Aβ epitopes. The 4G8 antibody binds to residues 17–24 of Aβ, which inhibits fibrinogen from binding to Aβ and thus prevents Aβ42/fibrinogen complex formation.^39^

The mOC64 anti‐Aβ antibody targets the N‐terminus of the Aβ peptide (residues 3–6) and therefore does not interfere with Aβ/fibrinogen complex formation. 4G8 prevented Aβ42/fibrinogen‐mediated synaptotoxicity in OHCs as evidenced by the lack of significant changes in SYP and PSD‐95 proteins compared to control (Figure 1D,F; blue vs. open circles), whereas mOC64 had no effect on Aβ42/fibrinogen‐mediated synaptotoxicity (Figure S1; blue vs. red circles). These findings provide evidence that the interaction between Aβ42 oligomers and fibrinogen is necessary to induce direct hippocampal synapse loss, supporting the role of this complex in AD‐related synaptic dysfunction.

### 3.2 The Aβ42/fibrinogen complex leads to a synergistic reduction in synapse proteins and an increase in tau phosphorylation in vivo

**FIGURE 2:**
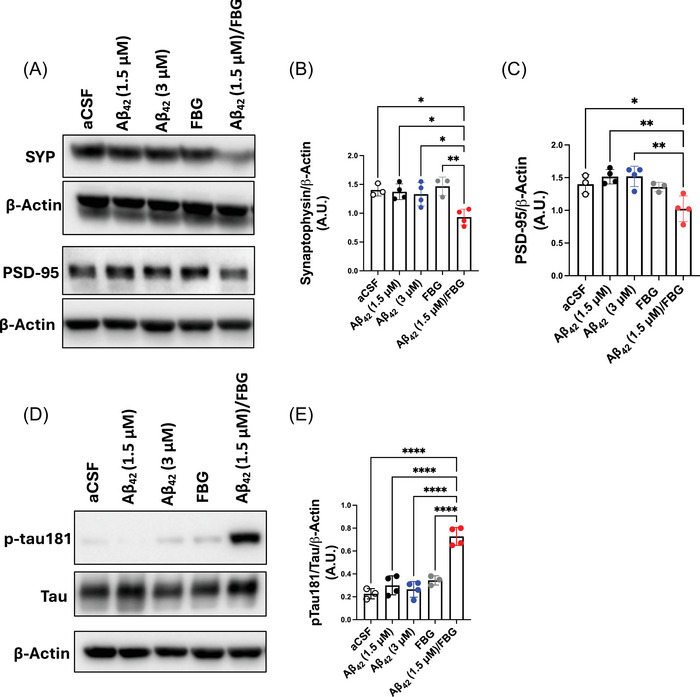
The Aβ42/fibrinogen complex reduces SYP and PSD‐95 levels and induces tau181 phosphorylation (p‐tau181) in the hippocampus of WT mice. WT mice were injected ICV with aCSF, Aβ42 (1.5 or 3.0 µM), fibrinogen (FBG, 7.5 µM), or the preformed Aβ42/FBG complex using low‐dose (1.5 µM) Aβ42. Three days post‐injection, brains were processed for Western blotting. (A) Representative Western blot of SYP and PSD‐95 levels in hippocampal homogenates. Protein analyses were normalized to *β*‐actin. *β*‐actin bands shown are the same as in Figure 5F; this membrane and sample set was probed for multiple proteins. Quantification shows a reduction of (B) SYP and (C) PSD‐95 in hippocampal homogenates of mice injected with the Aβ42/FBG complex compared to other treatments (red). (D) Representative Western blot of total tau and phosphorylated tau181 (p‐tau181) levels in the same hippocampal homogenates. (E) Quantification of Western blots indicates that ICV injection of the Aβ42/FBG complex led to increased tau181 phosphorylation in the WT mouse hippocampus compared to other treatments (red). Statistical analyses were performed using one‐way ANOVA followed by Tukey's post‐hoc test. Bar graphs represent mean ± SEM. *n* = 3–4 mice/group. Aβ, amyloid‐beta; aCSF, artificial cerebrospinal fluid; ANOVA, analysis of variance; ICV, intracerebroventricularly; PSD, postsynaptic density protein; SEM, structural equation modeling; SYP, synaptophysin; WT, wild‐type. ^*^ *p* < 0.5, ^**^ *p* < 0.01, ^****^ *p* < 0.0001

To determine if the Aβ42/fibrinogen complex could also induce hippocampal synaptotoxicity in vivo, intracerebroventricular (ICV) injections of Aβ42 and/or fibrinogen were performed in adult C57BL/6 (WT) mice. Hippocampi were collected 3 days post‐injection and analyzed biochemically. Mice that received the Aβ42/fibrinogen complex exhibited a significant reduction in SYP and PSD‐95 protein content in the hippocampus, while administration of singular Aβ42 (1.5 or 3.0 µM) or fibrinogen (7.5 µM) did not induce significant changes in these synapse protein levels (Figure 2A–C).

To determine the relevance of the Aβ42/fibrinogen complex on mediating synaptotoxicity in vivo, we used the 4G8 anti‐Aβ antibody to block complex formation as described above.

Consistent with the OHC results, SYP and PSD‐95 levels from hippocampi of the [(Aβ42 + 4G8) + FBG]‐injected group showed no significant difference when compared to the aCSF control group (Figure S2; blue vs. open circles). Therefore, blocking complex formation protected against Aβ42/fibrinogen‐induced synaptotoxicity in vivo.

Another pathological hallmark of AD is hyperphosphorylated tau (p‐tau). In post‐mortem AD brain analyses, various p‐tau species, including p‐tau at amino acid 181 (p‐tau181), are present in pretangles and NFTs.^40^, ^41^ In preclinical AD, when there are only subtle changes in brain Aβ levels, p‐tau181 levels increase in the CSF and plasma, suggesting that p‐tau181 could be used as an early AD biomarker.^33^, ^42^ Moreover, a study using *App* knock‐in mice showed a relationship between various p‐tau species (p‐tau181, p‐tau217, and p‐tau231) and brain Aβ accumulation, which suggested that plasma p‐tau181 could be indicative of brain Aβ pathology before pretangle or NFT formation.^43^ Therefore, we examined our samples for the presence of p‐tau181 in hippocampal tissue as an indicator of early synaptic pathology in these ICV‐injected WT mice. While mouse p‐tau is not considered as neurotoxic as human p‐tau, it has been linked to postsynaptic AD pathology in various mouse models. In our studies, Western blot analysis revealed a significant increase in hippocampal p‐tau181 expression in mice injected with the pre‐formed Aβ/fibrinogen complex compared to vehicle or individual Aβ42 (1.5 or 3.0 µM) or fibrinogen (7.5 µM) treatments (Figure 2D,E). These findings suggest an important role of the Aβ42/fibrinogen complex in inducing hippocampal synaptotoxicity and early tau phosphorylation in vivo, which could be a contributing pathological factor in the early stages of AD.

### 3.3 The Aβ42/fibrinogen complex leads to early astrogliosis and microgliosis in vivo

**FIGURE 3:**
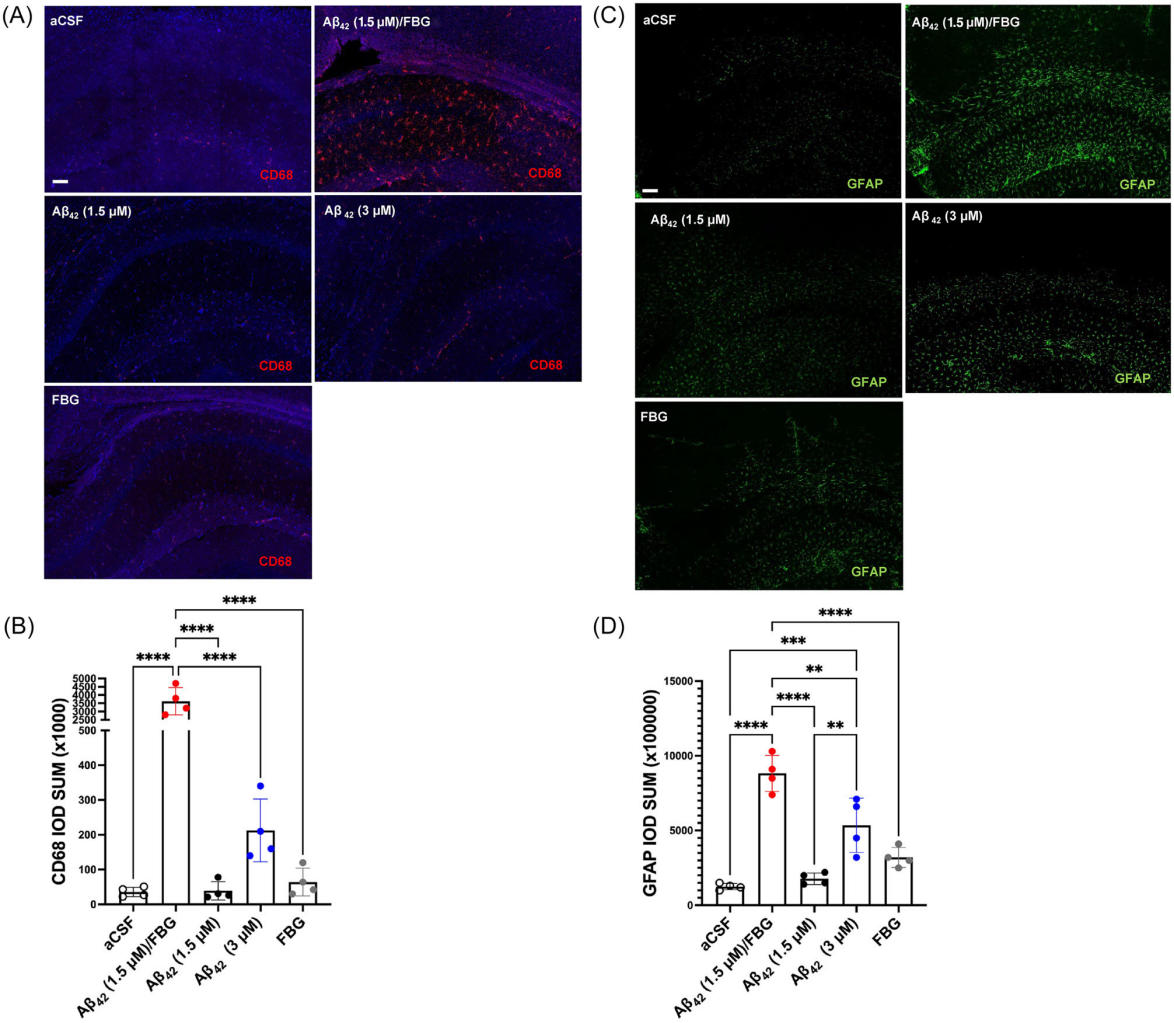
The Aβ42/FBG complex increases inflammatory activity in the hippocampus of WT mice. WT mice were injected ICV with aCSF, the pre‐formed Aβ42/FBG complex (1.5 or 7.5 µM, respectively), Aβ42 oligomers (1.5 or 3.0 µM), or FBG (7.5 µM). After 3 days, brains were processed for immunostaining. Hippocampal sections were stained for (A) CD68 (red) or (B) GFAP.^70^ (C), (D) There was significantly more CD68 and GFAP staining in the CA1 region of the hippocampus of the Aβ42/FBG complex‐injected mouse group compared to all other treatment groups. Scale bar, 50 µm. Data were analyzed by one‐way ANOVA with Tukey's post hoc analysis. *n* = 4/group. Aβ, amyloid‐beta; aCSF, artificial cerebrospinal fluid; ANOVA, analysis of variance; FBG, fibrinogen; GFAP, glial fibrillary acidic protein; ICV, intracerebroventricularly; WT, wild‐type. ^**^ *p* < 0.01, ^***^ *p* < 0.001, ^****^ *p* < 0.0001.

It has been shown that Aβ oligomers and/or fibrinogen injected into the ventricles of the mouse brain can induce inflammatory cell activation.^11^, ^22^, ^23^ In order to determine if ICV injection of the Aβ42/fibrinogen complex could also trigger or enhance this inflammatory phenotype in vivo, we examined expression of CD68, a transmembrane protein expressed by phagocytic microglia, infiltrating monocytes, and macrophages, as well as GFAP, an astrocytic marker, via immunofluorescence of the mouse hippocampus. Three days after ICV injection, hippocampal slices from mice injected with the Aβ/fibrinogen complex exhibited a significant increase in CD68 and GFAP immunoreactivity in the CA1 region of the hippocampus (Figure 3). However, WT mice injected with vehicle or Aβ42 or fibrinogen alone failed to induce significant gliosis in the hippocampus (Figure 3).

### 3.4 The Aβ42/fibrinogen complex induces early synaptic loss independent of the Mac‐1 receptor in vitro and in vivo

**FIGURE 4:**
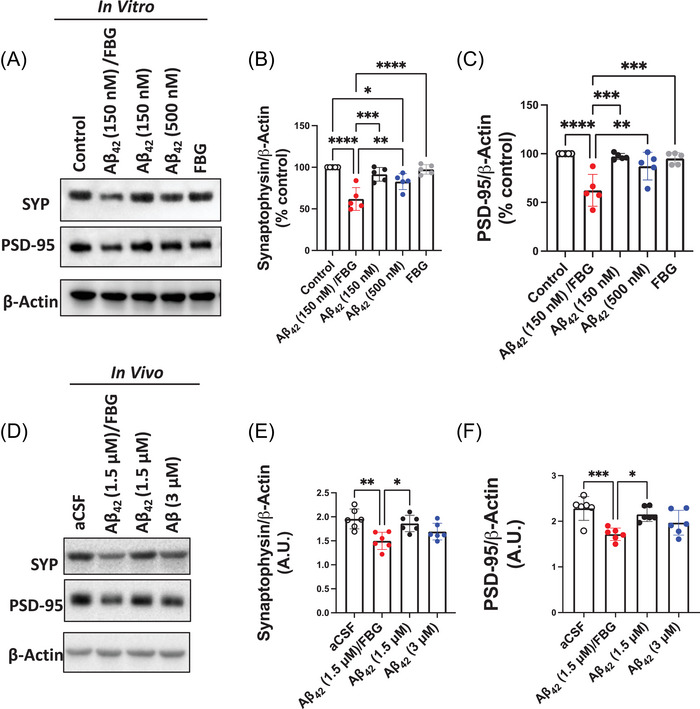
The Aβ42/fibrinogen complex induces synaptotoxicity independent of Mac‐1 receptor signaling. (A)–(C) OHC slices from Mac‐1 KO mice were incubated with vehicle (0.005% DMSO in PBS; control), high or low doses of Aβ42 oligomers (500 or 150 nM), FBG (50 nM), or the pre‐formed Aβ42/FBG complex (150 and 50 nM, respectively) for 24 h. Following treatment, OHCs were washed, lysed, and prepared for biochemical analysis. (A) Representative Western blot of SYP and PSD‐95 levels in Mac‐1 KO OHC lysate. Quantification shows a reduction of (B) SYP and (C) PSD‐95 in Mac‐1 KO slices treated with Aβ42/FBG complex only (red). *n* = 5 independent experiments/5 wells per treatment; each experiment utilized 3 mice; 15 mice in total. (D)–(F) Mac‐1 KO mice were injected ICV with aCSF, Aβ42/FBG complex (1.5 and 7.5 µM, respectively), Aβ42 oligomer (1.5 or 3.0 µM), or FBG (7.5 µM). Three days post‐injection, brains were processed for Western blotting. (D) Representative Western blot of SYP and PSD‐95 levels in hippocampal homogenates. Protein analyses were normalized to β‐actin. Quantification shows a reduction of (E) SYP and (F) PSD‐95 in hippocampal homogenates from mice injected with Aβ42/FBG complex (red). *n* = 5 mice/group. Statistical analyses were performed using one‐way ANOVA followed by Tukey's post‐hoc test. Aβ, amyloid‐beta; aCSF, artificial cerebrospinal fluid; ANOVA, analysis of variance; DMSO, dimethyl sulfoxide; FBG, fibrinogen; ICV, intracerebroventricularly; KO, knockout; OHC, organotypic hippocampal slice culture; PBS, phosphate buffered saline; PSD, postsynaptic density protein; SYP, synaptophysin; WT, wild‐type. ^*^ *p* < 0.05, ^**^ *p* < 0.01, ^***^ *p* < 0.001,^****^ *p* < 0.0001.

Fibrinogen binds to the Mac‐1 (CD11b‐CD18) integrin receptor on microglia, inducing pathogenic microglial activation. This receptor binding also leads to spine elimination in a mouse model of AD.^23^ Our results suggested an increase in phagocytic activity of microglia/infiltrating monocytes and macrophages based on the increase in hippocampal CD68 staining upon Aβ42/fibrinogen treatment (Figure 3). Therefore, we investigated if the Mac‐1 receptor plays a role in Aβ42/fibrinogen complex‐mediated synaptotoxicity. To explore this mechanism, we used the same in vitro and in vivo techniques described above using mice lacking Mac‐1 (Mac‐1 KO). Similar to the in vitro results with WT mice, OHCs derived from Mac‐1 KO mice exhibited a significant reduction in SYP and PSD‐95 synaptic proteins following treatment with the Aβ42/fibrinogen complex (Figure 4A–C; red vs. open circles). In line with the in vitro Mac‐1 KO OHC findings, ICV injection of the Aβ42/fibrinogen complex into Mac‐1 KO mice resulted in a reduction of synaptic proteins three days after administration (Figure 4D–F; red vs. open circles). This decrease was observed exclusively in the group that received the Aβ42/fibrinogen complex, emphasizing its distinct impact compared to the individual administration of Aβ42 oligomers or fibrinogen. Additionally, Mac‐1 KO mice showed a significant increase in hippocampal p‐tau181 levels exclusively in the group that received the injection of Aβ42/fibrinogen complexes (Figure S3). Taken together, these findings suggest that the Aβ42/fibrinogen complex induces synaptotoxicity through a Mac‐1‐independent pathway.

### 3.5 The Aβ42/fibrinogen complex leads to BBB disruption in vivo

**FIGURE 5:**
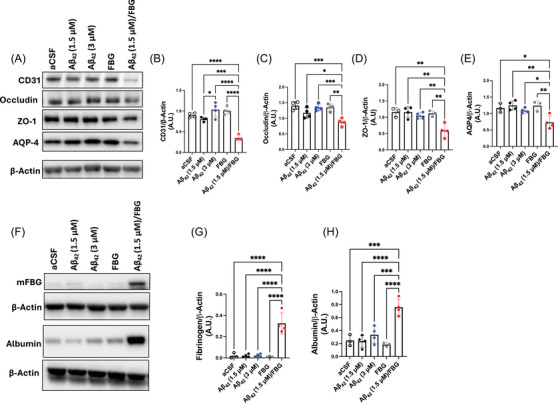
The Aβ42/FBG complex induces BBB disruption and extravasation in the hippocampus. WT mice were ICV injected with aCSF, Aβ42 /FBG complex (1.5 and 7.5 µM, respectively), Aβ42 oligomers (1.5 or 3.0 µM), or FBG (7.5 µM). Three days post‐injection, brains were processed for Western blotting. Hippocampal homogenates were analyzed by Western blot for CD‐31, occludin, ZO‐1, and AQP‐4. (A) Representative Western blot. (B)–(E) Quantification of CD31, occludin, ZO‐1, and AQP‐4 levels in hippocampal homogenates, normalized to β‐actin. The Aβ42/FBG complex led to a synergistic decrease in all proteins examined (red). (F) Representative Western blot of hippocampal homogenates probed for mouse fibrinogen and albumin. *β‐*actin bands shown are the same as in Figure 2A; this membrane and sample set was probed for multiple proteins. (G), (H) Quantification of fibrinogen and albumin normalized to *β‐*actin. The Aβ42/FBG complex induced significantly more extravasation of blood proteins into the mouse hippocampus compared to all other treatment groups (red). Statistical analyses were performed using one‐way ANOVA followed by Tukey's post‐hoc test. Bar graphs represent mean ± SEM. Aβ, amyloid‐beta; aCSF, artificial cerebrospinal fluid; ANOVA, analysis of variance; BBB, blood–brain barrier; FBG, fibrinogen; ICV, intracerebroventricularly; PSD, postsynaptic density protein; SEM, structural equation modeling; SYP, synaptophysin; WT, wild‐type. *n* = 3–4/group. ^*^ *p* < 0.05, ^**^ *p* < 0.01, ^***^ *p* < 0.001, ^****^ *p* < 0.0001.

BBB disruption and the subsequent extravasation of plasma proteins from the vessels into the brain parenchyma is a potential contributor to the progression of AD.^12^, ^20^ To assess the impact of the Aβ42/fibrinogen complex on BBB integrity following ICV injection, we analyzed several neurovascular unit markers, including CD31, occludin, ZO‐1, and aquaporin‐4 (AQP‐4). Hippocampal homogenates from mice injected with Aβ42/fibrinogen complex were compared to control groups. Our results showed a significant decrease in levels of CD31, occludin, ZO‐1, and AQP‐4 in hippocampal samples from the Aβ42/fibrinogen complex‐injected group compared to all other treatment groups (Figure 5A–E; red circles). These findings support the hypothesis that the Aβ42/fibrinogen complex is involved in the loss of BBB integrity at the level of endothelial cells, tight junctions, and astrocytic endfeet.

To validate the association between the observed decrease in neurovascular unit‐related proteins and BBB breakdown, we evaluated the extravasation of mouse fibrinogen and albumin into the WT mouse hippocampus after ICV administration of Aβ42, fibrinogen, or its complex. Consistent with our previous results,^20^, ^44^ there was significantly more extravasated mouse fibrinogen and albumin upon Aβ42/fibrinogen complex administration compared to other groups (Figure 5F–H; red circles). These data confirm BBB damage and plasma protein extravasation upon indirect Aβ42/fibrinogen exposure.

Overall, our study provides evidence that the Aβ42/fibrinogen complex can induce BBB damage, enhancing neuroinflammation and synaptotoxicity in the brain that could be critical in the pathogenesis of AD.

## 4 DISCUSSION

Fibrinogen can bind to the central region of the Aβ peptide, forming plasmin‐resistant and morphologically abnormal clots.^25^, ^26^, ^39^ This process leads to persistent fibrin deposition, which can contribute to an enhanced neuroinflammatory and degenerative process.^20^, ^45^ However, the direct contribution of the Aβ/fibrinogen complex to synaptic damage and neuroinflammation is still unclear. In this study, we utilized in vitro and in vivo models to investigate the impact of the Aβ42/fibrinogen complex on synapse degeneration, neuroinflammation, BBB damage, and early tau pathology in adult WT mice. Our results demonstrate that the Aβ42/fibrinogen complex has a synergistic impact on hippocampal synaptotoxicity, neuroinflammation, and BBB disruption. This synergistic effect was dependent on the Aβ42/fibrinogen interaction, as administration of Aβ42 in high or low doses or fibrinogen alone or preventing the binding of Aβ42 and fibrinogen and thus the complex from forming, did not induce these pathologies. Furthermore, Aβ42/fibrinogen complexes functioned independently of microglial fibrinogen receptor Mac‐1 signaling. Interestingly, ICV administration of the Aβ42/fibrinogen complex also led to early tau phosphorylation in the hippocampus, suggesting that the presence of this complex may serve as a link between Aβ and early tau pathologies in AD.

The hippocampus, crucial for learning and memory processes, is one of the earliest brain regions affected in AD with the number of synaptic connections lost corresponding with the progression of cognitive decline.^1^, ^46^ Consistent with previous findings,^30^ our results show that Aβ42 oligomers dose‐dependently induce synaptic protein loss in WT mouse OHCs (Figure 1A–C), mirroring AD‐like synaptic dysregulation. Other studies have suggested that fibrinogen itself can induce dendritic spine elimination in the regions of their own parenchymal deposits.^22^, ^23^, ^31^ However, we did not observe significant synaptic loss in fibrinogen‐treated OHCs or ICV‐injected mice (Figures 1 and 2). This disparity may be explained by the use of lower and more physiologically relevant fibrinogen concentrations and/or differences in brain injection sites. In previous studies,^22^, ^23^, ^47^ fibrinogen was directly injected into the brain parenchyma, whereas we administered it into the brain ventricular system via the right lateral ventricle. The ventricular system allows for widespread effects of injected substances rather than direct, targeted administration to a region of interest in the brain. Our Western blot results were obtained from the hippocampi that were contralateral to the injected lateral ventricle, further ensuring that any observed changes in synaptotoxicity were due to physiological spreading of the administered substances and not due to direct administration into a brain region.

Here, we found that the Aβ42/fibrinogen complex showed a pathological effect on synaptic damage at low concentrations, both in vitro and in vivo, whereas at low concentrations, treatment with fibrinogen or Aβ42 alone did not induce synaptotoxicity. We confirmed that the mechanism underlying increased synaptotoxicity upon co‐treatment with Aβ42 oligomers and fibrinogen (the pre‐formed Aβ42/fibrinogen complex) was attributed to their interaction, as blocking the fibrinogen binding site on Aβ42 using a neutralizing antibody, 4G8, prevented this synaptic damage in vitro and in vivo (Figure 1D–F and Figure S2). Using a different anti‐Aβ antibody that does not block Aβ/fibrinogen binding (mOC64) did not affect the observed Aβ/fibrinogen‐induced synaptotoxocity (Figure S1).

These results highlight the importance of Aβ42/fibrinogen complex formation in AD synaptic dysfunction. These findings align well with clinical observations of fibrinogen extravasation and its colocalization with Aβ plaques,^32^, ^48^, ^49^, ^50^ suggesting that fibrinogen within plaques may exacerbate synaptotoxicity in AD patient brains.

Another key pathological feature of AD is the accumulation of phosphorylated tau, which is also associated with synaptic impairment, neuronal dysfunction, and the onset of dementia.^2^, ^33^, ^51^, ^52^ Our results show that administration of the Aβ42/fibrinogen complex was able to induce tau181 phosphorylation in vivo, whereas Aβ42 and fibrinogen alone did not (Figure 2D,E). Previous studies have suggested that Aβ42 is able to induce tau phosphorylation; however, the Aβ42 concentration required to achieve this in mice was higher than the concentrations used in our study.^53^, ^54^ Nonetheless, p‐tau181 is an established and widely used plasma biomarker that identifies individuals at risk of cognitive decline and/or in their early stages of AD.^33^, ^55^ Our data suggest that the interaction between Aβ42 oligomers and fibrinogen may be a contributing factor to early stages of tau pathology in AD.

Microglia and astrocytes play a pivotal role in trying to protect the brain from AD pathology, releasing protective substances and/or clearing toxic materials around surrounding neurons.^6^, ^10^, ^56^ A study found that fibrinogen at sites of vascular damage can induce neuroinflammation by binding to the Mac‐1 receptor on microglia, consequently activating a pathogenic cascade and promoting spine loss and cognitive decline in mouse models.^23^ Preventing fibrinogen from interacting with the Mac‐1 receptor lowered neuroinflammatory activity, synaptic dysfunction, and cognitive decline in an AD mouse line.^23^ Our results revealed that only the complex of Aβ42 and fibrinogen (not the individual proteins) induced robust activation of microglia and astrocytes, implicating these inflammatory responses in mediating the loss of synaptic proteins. Importantly, when Mac‐1 KO mice were subjected to the same in vivo approach, the Aβ42/fibrinogen complex was just as synaptotoxic as observed in WT mice (Figure 4). Thus, our findings suggest that Aβ42/fibrinogen complex‐mediated synaptotoxicity in the brain is independent of fibrinogen‐mediated Mac‐1 receptor signaling.

Aβ and fibrinogen can interact with numerous receptors and proteins. For instance, Aβ and fibrinogen can bind to ICAM‐1,^57^, ^58^ which can regulate cellular responses to inflammation and injury. ICAM‐1 acts as a biosensor to induce outside‐in‐signaling via its association with actin.^59^, ^60^ It is noteworthy that AD brains show increased expression of ICAM‐1 in astrocytes surrounding Aβ plaques,^61^, ^62^ suggesting that ICAM‐1 could be a potential downstream target of the Aβ42/fibrinogen complex. Another possible mechanistic pathway could involve apolipoprotein E (ApoE), which interacts with Aβ and regulates its accumulation and clearance from the brain. ApoE has also been linked to BBB disruption in AD.^63^ Therefore, further detailed studies will be necessary to fully understand the mechanism(s) by which the Aβ42/fibrinogen complex induces synaptotoxicity and neuroinflammation in AD pathophysiology.

Studies have shown that both normal aging and neurodegenerative processes involve BBB dysfunction, contributing to the extravasation of plasma proteins, such as thrombin, fibrinogen, albumin, and immunoglobulin G (IgG), into the brain parenchyma.^14^, ^64^, ^65^, ^66^ Various human and animal studies found that fibrinogen is often associated with amyloid plaques, neurovascular damage, and neuroinflammation.^12^, ^19^, ^21^, ^66^ Interestingly, BBB damage has been identified within the hippocampus in preclinical AD, independent of Aβ and tau pathological progression.^67^, ^68^ In accordance with animal and clinical studies, we demonstrate here that the Aβ42/fibrinogen complex can induce BBB deficits as evidenced by decreased expression of neurovascular unit markers in endothelial cells, tight junctions, and astrocytic endfeet in addition to fibrinogen and albumin extravasated into the hippocampus (Figure 5).

Overall, our study elucidates the multifaceted roles of the Aβ42/fibrinogen complex in driving synaptic dysfunction, neuroinflammation, and BBB disruption in AD pathophysiology. By unraveling the intricate interplay between these pathological factors, our findings provide novel insights into early AD pathogenesis and identify potential therapeutic targets for intervention. While our study was limited in scope by only utilizing male subjects for our studies, hormonal responses are minimal between sexes at the ages of mice used in our experiments.^69^ However, repeating these studies in older male and female mice would be helpful in determining if sex is a biological factor in Aβ42/fibrinogen‐induced synaptotoxicity. Furthermore, our research focused specifically on understanding the impact of the Aβ42/fibrinogen complex on synaptotoxicity, neuroinflammation, and BBB integrity, so future research is needed to explore the effects of the Aβ42/fibrin complex on these pathologies. Continued studies are also necessary to elucidate the precise downstream mechanisms underlying the synergistic effects of Aβ42 and fibrinogen and to validate therapeutic potential in both preclinical and clinical settings. Overall, our findings suggest that inhibiting Aβ42/fibrinogen complex formation could be a novel therapeutic target for mitigating neuroinflammation and synaptotoxicity in AD.

## AUTHOR CONTRIBUTIONS

Elisa Nicoloso Simões‐Pires designed the study, performed experiments, analyzed data, and wrote the manuscript. Daniel Torrente performed experiments, participated in data analysis, and wrote the manuscript. Pradeep Singh aided in study design and data analysis. Sidney Strickland participated in data analysis and reviewed the manuscript. Erin H. Norris designed the study, participated in data analysis, and wrote the manuscript.

## CONFLICT OF INTEREST STATEMENT

The authors declare no conflicts of interest. Author disclosures are available in the supporting information.

## CONSENT

No human subjects or clinical work, so consent is not applicable.

## Supporting information



Supporting Information

Supporting Information
