# Supplementary material for: Synergistic effects of the Aβ/fibrinogen complex on synaptotoxicity, neuroinflammation, and blood–brain barrier damage in Alzheimer's disease models
Source: Alzheimers Dement. 2025 May 8;21(5):e70119. doi: 10.1002/alz.70119 (PMC12061846; doi:10.1002/alz.70119)
Supplement: Supplementary file 1 — Supporting Information [file ALZ-21-e70119-s002.docx]

**Supplementary material for**

**Synergistic effects of the Aβ/fibrinogen complex on synaptotoxicity, neuroinflammation, and blood-brain barrier damage in Alzheimer’s disease models**

Elisa Nicoloso Simões-Pires^1^, Daniel Torrente^1^, Pradeep Singh^1^, Sidney Strickland^1^, and Erin H. Norris^1a^

^1^Patricia and John Rosenwald Laboratory of Neurobiology and Genetics, The Rockefeller University, New York, NY 10065, USA

^a^To whom correspondence should be addressed: [enorris@rockefeller.edu](mailto:enorris@rockefeller.edu)


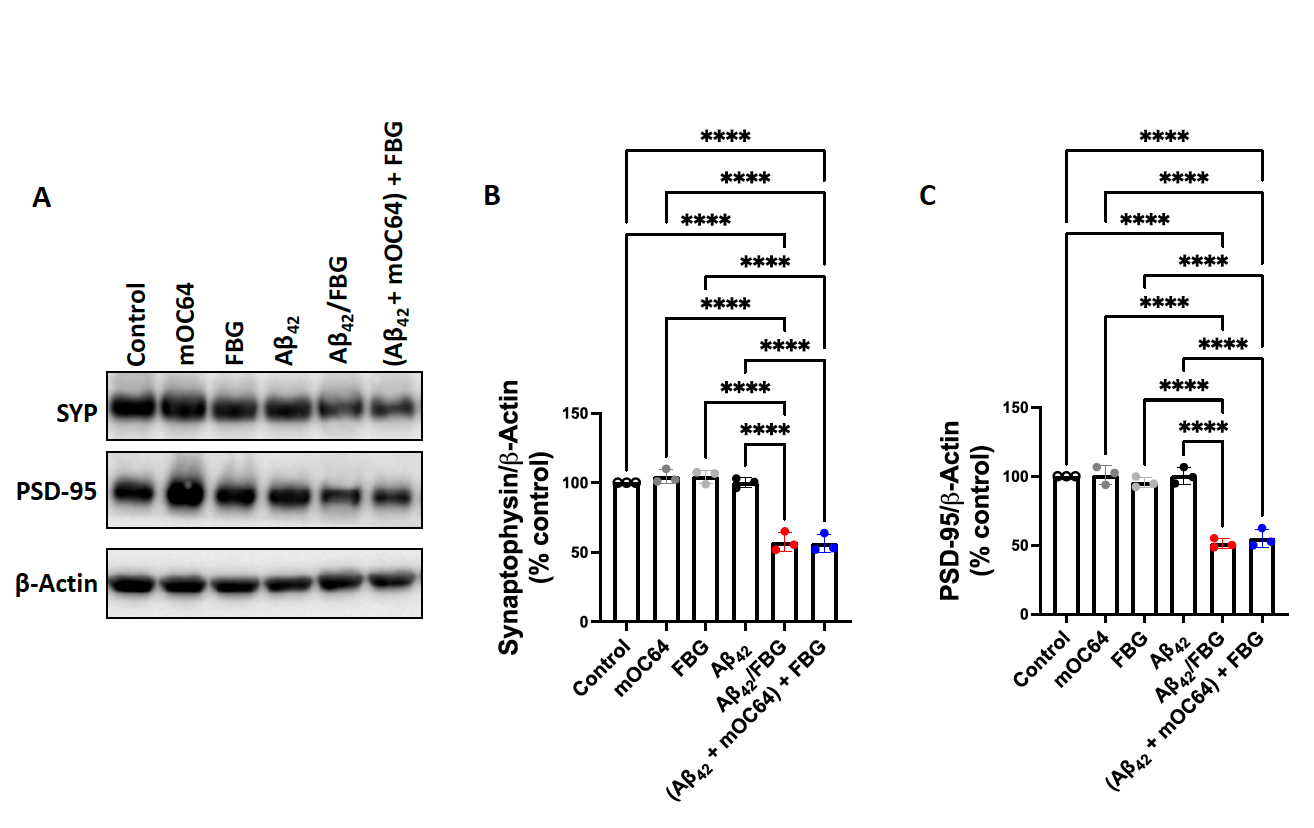


**Supplementary Figure 1. The Aβ_42_/fibrinogen complex induces synergistic synaptotoxicity in mouse organotypic hippocampal slice cultures (OHCs).** OHCs were treated with vehicle, low dose Aβ**_42_** oligomers (150 nM), FBG (50 nM), or Aβ**_42_**/FBG complex with or without mOC64 anti-Aβ antibody for 24 hours and then collected and prepared for biochemical analysis. **(A)** Representative Western blot of SYP and PSD-95 levels in OHC lysates. **(B, C)** Quantification of SYP and PSD-95 levels from OHC homogenates showed that mOC64, which does not block the Aβ_42_/fibrinogen interaction, did not prevent the synaptotoxicity induced by this complex (red and blue circles). Statistical analyses were performed using one-way ANOVA followed by Tukey’s post-hoc test. ****p<0.0001. Bar graphs represent mean ± SEM. n=3.


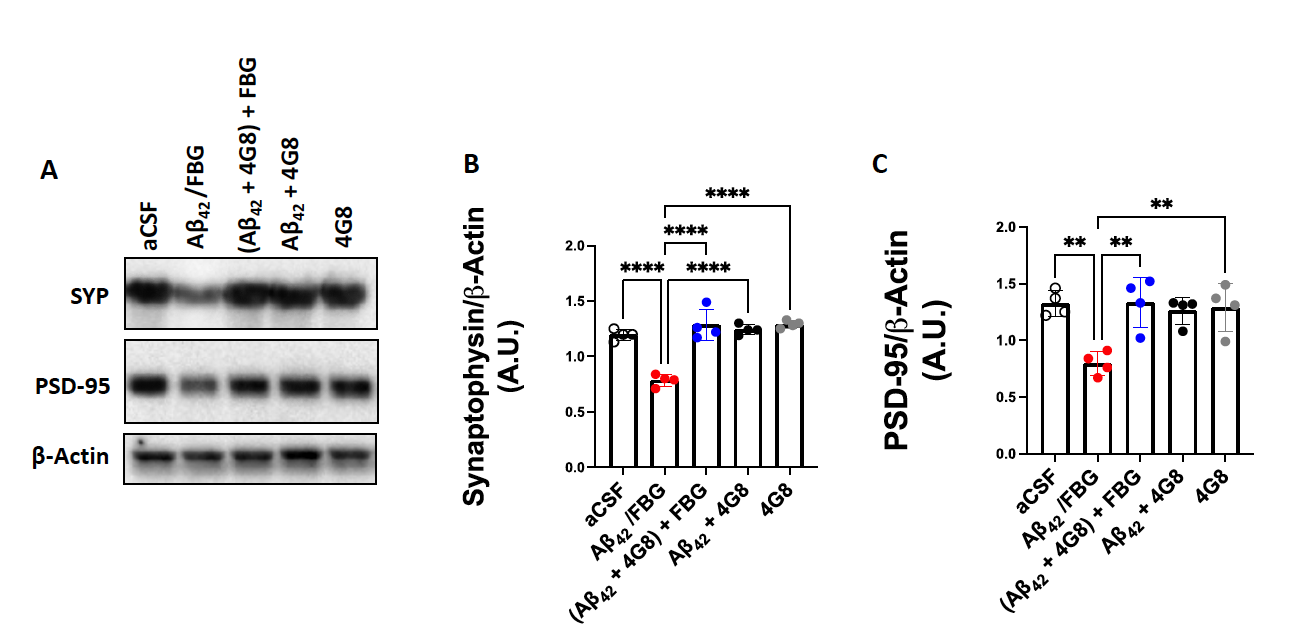


**Supplementary Figure 2. Blocking Aβ_42_/fibrinogen complex formation protects the mouse hippocampus from synaptotoxixicity *in vivo.*** The 4G8 anti-Aβ antibody was used to block Aβ_42_/fibrinogen complex formation prior to *in vivo* studies. WT mice were ICV-injected with aCSF, Aβ**_42_** /FBG complex (1.5 µM and 7.5 µM, respectively), [Aβ**_42_** (1.5 µM) + 4G8 (1.5 µM)] + FBG (7.5 µM), [Aβ**_42_** (1.5 µM) + 4G8 (1.5 µM)], or 4G8 (1.5 µM). Three days post-injection, mice were perfused and brains were dissected and processed. **(A)** Hippocampal homogenates were analyzed by Western blot for synaptic proteins, SYP and PSD-95. **(B, C)** Quantification of SYP and PSD-95 showed that blocking Aβ_42_/FBG complex formation with 4G8 prevented its synaptotoxicity in the hippocampi of WT mice (blue vs red circles). 4G8 alone or with Aβ42 had no effect on protein levels compared to aCSF control. Statistical analyses were performed using one-way ANOVA followed by Tukey’s post-hoc test. **p<0.01, ****p<0.0001. Bar graphs represent mean ± SEM. n=4 mice/group.

**
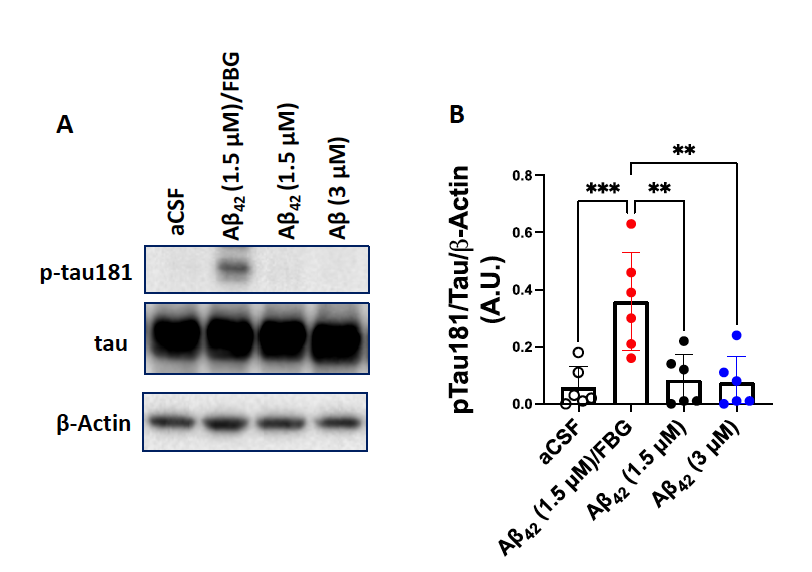
**

**Supplementary Figure 3. The Aβ_42_/fibrinogen complex induces tau181 phosphorylation in Mac-1 KO mouse hippocampus.** Mac-1 KO mice were ICV-injected with aCSF, Aβ**_42_**/FBG complex (1.5 µM and 7.5 µM, respectively), or Aβ**_42_** oligomers (1.5 µM or 3.0 µM). Three-days post-injection, brains were processed for Western blotting. **(A)** Hippocampal homogenates were analyzed by Western blot for p-tau181 and total tau levels. **(B)** Quantification showed a synergistic increase in p-tau181 in mouse hippocampi after ICV administration of Aβ_42_/FBG complex compared to other treatment groups (red circles). Statistical analyses were performed using one-way ANOVA followed by Tukey’s post-hoc test **p<0.01, ***p<0.001. Bar graphs represent mean ± SEM. n= 6 mice/group.


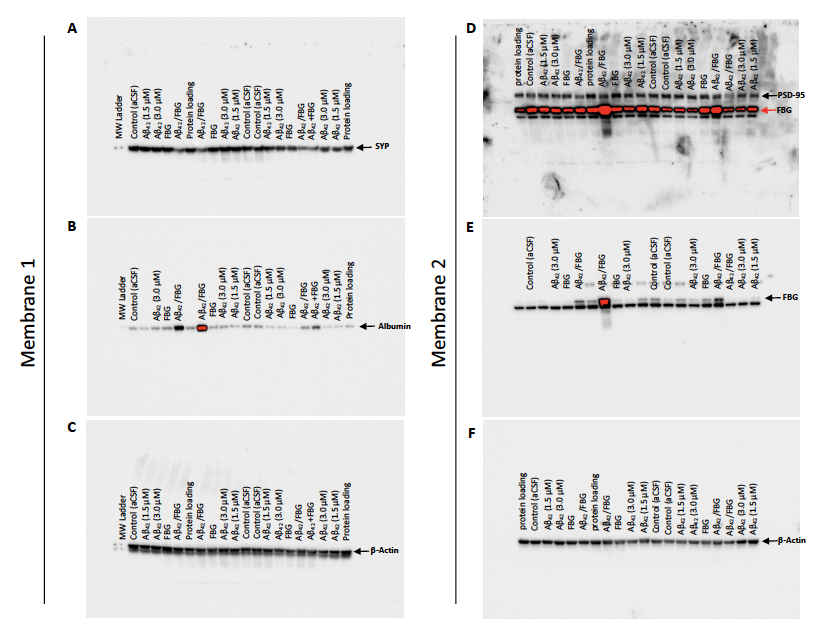


**Supplementary Figure 4. Uncropped gel membranes from Figures 2A and 5F.** Protein homogenates from *in vivo* experiments described in Figures 2 and 5 were run on two separate polyacrylamide gels, and then each membrane was probed for proteins of different molecular weights. Membrane 1 (A-C) was probed with antibodies against synaptophysin (SYP; A), albumin (B), and β-actin (C). Membrane 2 (D-F) was probed with antibodies against PSD-95 (D), fibrinogen (FBG; D, E), and β-actin (F). The antibodies were incubated separately, without stripping, on each membrane, and protein bands (black arrows) were viewed at different exposure times. β-actin from each respective membrane was used to normalize probed proteins for quantification. Red arrow in D indicates FBG at a longer exposure time when previously probed for PSD-95. β-actin bands are the same in Figures 2A and 5F since the proteins of interest are from the same Western blot membranes.
